# The Association between Preterm Birth and Ambient Air Pollution Exposure in Shiyan, China, 2015–2017

**DOI:** 10.3390/ijerph18084326

**Published:** 2021-04-19

**Authors:** Qihao Chen, Zhan Ren, Yujie Liu, Yunfei Qiu, Haomin Yang, Yuren Zhou, Xiaodie Wang, Kuizhuang Jiao, Jingling Liao, Lu Ma

**Affiliations:** 1 Department of Healthcare Management, School of Health Sciences, Wuhan University, Wuhan 430072, China; chenqihao@whu.edu.cn (Q.C.); zhanren@whu.edu.cn (Z.R.); liuYJstu@whu.edu.cn (Y.L.); qiuyunfei@whu.edu.cn (Y.Q.); haominyang@whu.edu.cn (H.Y.); ZhouYuren@whu.edu.cn (Y.Z.); xiaodiew@whu.edu.cn (X.W.); jiao_kui_zhuang@whu.edu.cn (K.J.); 2 Department of Nutrition and Food Hygiene, School of Public Health, Medical College, Wuhan University of Science and Technology, Wuhan 430081, China

**Keywords:** air pollution, preterm birth, early term birth

## Abstract

Shortening of the gestational duration has been found associated with ambient air pollution exposure. However, the critical exposure windows of ambient air pollution for gestational duration remain inconsistent, and the association between ambient air pollution and early term births (ETB, 37 to 38 weeks) has rarely been studied relative to preterm births (PTB, 28–37 weeks). A time-series study was conducted in Shiyan, a medium-sized city in China. Birth information was collected from the Shiyan Maternity and Child Health Hospital, and 13,111 pregnant women who gave birth between 2015 and 2017 were included. Data of the concentrations of air pollutants, including PM_10_, PM_2.5_, NO_2_, and SO_2_ and meteorological data, were collected in the corresponding gestational period. The Cox regression analysis was performed to estimate the relationship between ambient air pollution exposure and the risk of preterm birth after controlling the confounders, including maternal age, education, Gravidity, parity, fetal gender, and delivery mode. Very preterm birth (VPTB, 28–32 weeks) as a subtype of PTB was also incorporated in this study. The risk of VPTB and ETB was positively associated with maternal ambient air pollution exposure, and the correlation of gaseous pollutants was stronger than particulate matter. With respect to exposure windows, the critical trimester of air pollutants for different adverse pregnancy outcomes was different. The exposure windows of PM_10_, PM_2.5_, and SO_2_ for ETB were found in the third trimester, with HRs (hazard ratios) of 1.06 (95%CI: 1.04, 1.09), 1.07 (95%CI: 1.04, 1.11), and 1.28 (95%CI: 1.20, 1.35), respectively. However, for NO_2_, the second and third trimesters exhibited similar results, the HRs reaching 1.10 (95%CI: 1.03, 6.17) and 1.09 (95%CI: 1.03,1.15), respectively. This study extends and strengthen the evidence for a significant correlation between the ambient air pollution exposure during pregnancy and the risk of not only PTB but, also, ETB. Moreover, our findings suggest that the exposure windows during pregnancy vary with different air pollutants and pregnancy outcomes.

## 1. Introduction

Preterm birth (PTB, <37 weeks of gestation) is the second most common reason that results in neonatal death [1]. Over 15 million premature babies are born worldwide each year, accounting for 10% of the yearly total births. In China, about 1.2 million premature babies are born every year, accounting for 12% of the global total. It is well-documented that PTB not only accounts for most of the perinatal mortality but also leads to lifelong morbidity, including a range of neurologic, pulmonary, and circulatory outcomes [2]. The current studies suggest that a variety of factors are related to PTB, such as age, pre-eclampsia, chronic infections, genetic predisposition, and socioeconomic and lifestyle factors [3], while more epidemiological research supports that ambient air pollution exposure may play an important role in the occurrence of premature birth [4].

Although the pathophysiology of preterm birth remains poorly understood, the negative effects of oxidative stress, inflammation, and DNA damage caused by air pollutants on adverse pregnancy outcomes have been widely supported by the existing evidence [5,6]. However, an association between exposure to ambient air pollution and the risk of adverse pregnancy outcomes is still unclear. The conclusion that particulate matter (PM_2.5_ and PM_10_) exposure increases the risk of preterm birth has a wide consensus, but the correlation intension description is difficult to unify (OR = 1.06~1.42, per 10 μg/m^3^) [7,8,9]. Reports regarding the association between gaseous pollutants and reproductive health are more controversial. Several studies have observed no statistically significant association between SO_2_ and NO_2_ exposure and the risk of adverse pregnancy outcomes [10,11], whereas some cohort studies and meta-analyses showed the opposite results (OR = 1.19~1.48, per 10 μg/m^3^) [12,13]. Studies around the world also show inconsistent descriptions of the relationship between air pollution and preterm birth [14]. Furthermore, the debate on the definition of the exposure window of various air pollutants is still ongoing [15].

Confounding factors such as the meteorological parameters, maternal health status [14], seasonal trend [16], and uncertainty of the exposure assessment [17] are considered to be the important reasons for the inconsistent results. Meanwhile, according to the government reports, the air quality in most Chinese cities is still not up to WHO standards, and the areas around Beijing and Western Xinjiang are heavily polluted, while the rate of PTB (about 6%) in developed cities in China is generally lower than that in small and medium-sized cities in the southwest and northeast (about 9–10%) [18], which indicates that the socioeconomic factors of the study area such as economic level, main industries, and pollutant sources may also be potential confounding factors. However, most of the studies in China focus on large cities, and the research of small and medium-sized cities has not been paid enough attention. In addition, according to the latest definition of the American College of Obstetricians and Gynecologists (ACOG), early term birth (ETB, birth between 37 and 38 weeks) shows a higher risk of adverse neonatal outcomes [19], but the association between ambient air pollution exposure and the risk of ETB is currently rarely studied.

In this study, to further explore the relationship between ambient air pollution exposure and adverse pregnancy outcomes in small and medium-sized cities in China, we selected Shiyan as the study site, which is a medium-sized nonprovincial city with low population mobility and regards the automobile industry as its pillar industry.

## 2. Materials and Methods

### 2.1. Study Site

Shiyan is a prefectural-level city located in Northwestern Hubei Province (N109°25′–N111°35′, E109°25′–E111°35′) with an area of 23,680 square kilometers and a total population of 3.46 million (2019). The yearly means for the relative humidity and temperature are 70–75% and 14–17 °C. Shiyan is one of the largest automotive industrial bases in China and is supported by ecological cultural tourism and the modern service industry.

According to the Shiyan Municipal Bureau of Statistics in 2019, Shiyan has a total population of 3.462 million, and the urban population accounts for 56.5% of the total. The city has a permanent resident population of 3.398 million and a small floating population, which ensures the stability of the population characteristics in the study.

### 2.2. Study Population

We enrolled parturient women between 1 January 2015 and 31 December 2017 at Shiyan Maternity and Child Health Hospital, which is the largest maternity and child health hospital in Shiyan, accounting for more than half of urban childbirths in Shiyan. The basic information of the delivery population was gained, including the basic information of the pregnant women, the childbirth information, and the infants’ information. Cases were excluded from the analysis if they lived out of the city, gave non-singleton births, had babies with extreme birth weights (<500 g or >5000 g), or gave birth at an extreme gestational age (<20 weeks or >42 weeks).

Gestational age at delivery (in weeks) was assessed by a midwife or obstetrician, who considered both the date of the last menstrual period and the ultrasound results during pregnancy. Discrepancies were resolved by clinical judgment. In addition to PTB and ETB, we further add very preterm births (VPTB, 28 to 32 weeks), one of the subtypes of PTB into this study.

The protocol in this study was approved by the Ethics Committee of Wuhan University (Project Identification Code 2020YF0020) and medical school district administrators.

### 2.3. Exposure Assessment

Daily average concentrations of PM_10_, PM_2.5_, NO_2_, and SO_2_ were obtained from four the air quality monitoring station (Liujiagou, Wudangshan, Tieerchu, and Binhexincun) from the Ministry of Ecology and Environment of the People’s Republic of China (http://106.37.208.233:20035/, 1 January 2015 and 31 December 2017). In order to explore the correlation between exposure to air pollutants and preterm births at different stages of pregnancy, we divided pregnancy into three exposure windows. Starting from the last menstrual period and ending from the date of delivery, we defined 0–12 weeks of pregnancy as the first trimester, 13–28 weeks as the second trimester, and from the 28th week to delivery day as the third trimester. An average ambient air pollution concentration was simulated for each participant in accordance with different exposure windows.

### 2.4. Statistical Analysis

Since pregnant women with different gestational ages may have different days of exposing periods, Cox proportional hazards regression models were selected to examine the associations between the mean concentrations of air pollutants at each stage of pregnancy and adverse birth outcomes. Survival time was defined as the duration from the first day of the last menstrual period to the date of birth. The mean concentrations of air pollutants during each stage of pregnancy (three trimesters and the whole pregnancy) were calculated for each participant to represent their trimester-specific exposures to ambient air pollution. We fitted gestational age as the time scale and defined spontaneous PTB, VPTB and ETB as the events.

According to the previous literature, the variables that were considered to have biological importance or potential confounding effects on preterm birth were included in the models: maternal age, maternal education, gravidity, parity, and fetal gender. Models were developed for each contaminant and each period of pregnancy (three trimesters and the whole pregnancy), respectively. We also conducted stratified analyses for the covariables that were considered as possible moderators, such as maternal age and education.

Considering that, the association between ambient air pollution and preterm birth is complex (nonlinear), and the exposure–response relationship was further checked by smoothing the air pollutant concentration terms using the splines function (with 3 df). R i386 3.5.2 software was used for data manipulation and statistical analysis, and the “survival” package was used for smoothing the curves, while the detailed information is available in the following package reference manual: https://cran.r-project.org/web/packages/survival/vignettes/splines.pdf (23 September 2019).

## 3. Results

### 3.1. Descriptive Analysis

**Figure 1:**
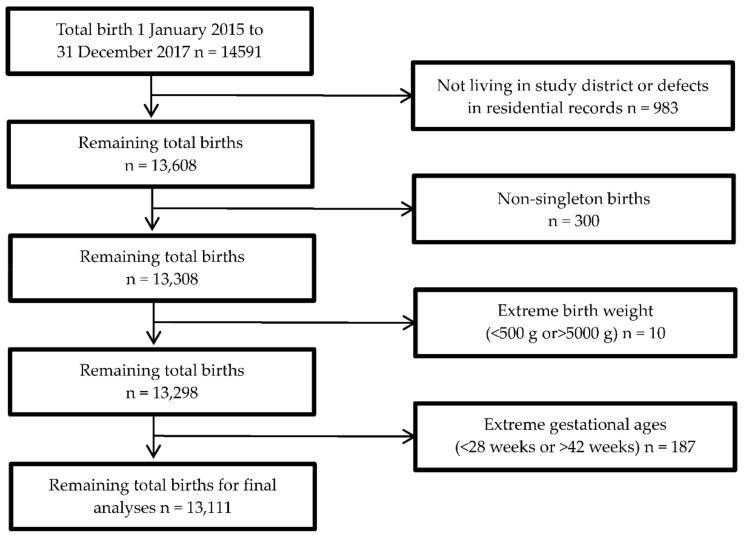
Exclusion process of the birth data used in the analyses.

A total of 14,591 births were recorded in the Hospital Information System (HIS) database, and 13,111 births were included in the final analysis after excluding 1480 cases: not living in the study district or defaults in the residential records (*n* = 983), twin pregnancies and multiple pregnancies (*n* = 300), birth records with extreme birth weight (*n* = 10), and extreme gestational age (*n* = 187) (Figure 1).

The characteristics of the study population are summarized in Table 1. Among the enrolled 13,111 singleton live births, 1246 (9.50%) were ETB and 614 (4.68%) were PTB, which included 63 (0.48%) VPTB. Most parturient women were under the children-bearing age (<35 years old, 89.76%), with high school degrees or above (84.10%), had more than once pregnancy (66.53%), and gave delivery for the first time (54.56%).

**Figure 2:**
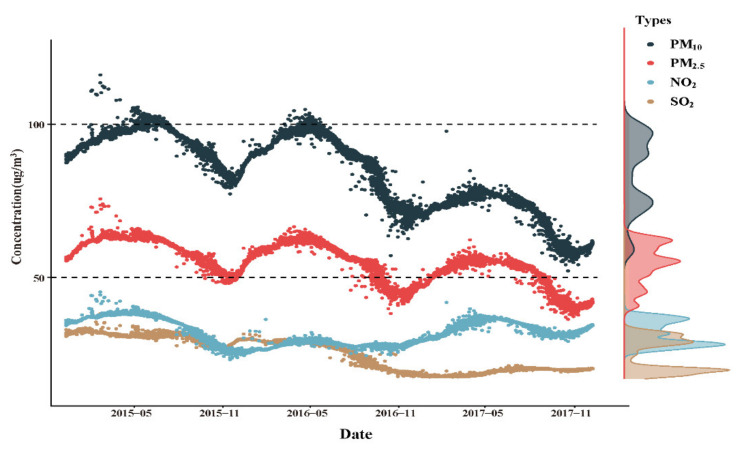
Ambient air pollution exposure to all participants, Shiyan, China, January 2015 to December 2017.

The change and trend of air pollutant concentration levels over time are displayed in Figure 2. The daily average concentrations for PM_2.5_, PM_10_, SO_2_, and NO_2_ were 50.14 µg/m^3^, 77.70 µg/m^3^, 20.37 µg/m^3^, and 29.17 µg/m^3^, respectively. Over the three-year study period, most air pollutants were on the decline, except NO_2_ (Figure 2). Each point represented the air pollutant concentration for a participant exposed throughout pregnancy. The results showed that the concentrations of PM_2.5_, PM_10_, SO_2_, and NO_2_ had obvious seasonal trends with the highest in the winter and the lowest in the summer. In this study, the number of births was not evenly distributed each year, and the concentration of air pollutants varies with the season, thus resulting in a bimodal distribution of exposure, as delineated in the right density curve of Figure 2. The detailed mean and standard deviation of the air pollutant exposure concentrations for the study population are presented in Table A1.

### 3.2. Statistical Model Analysis

After controlling the confounders, which included maternal age, education level, fetal gender, gravidity, and parity, we found that the ambient air pollution exposure was significantly associated with ETB and VPTB in the whole pregnancy (Table 2). The exposure windows of various air pollutants for different adverse pregnancy outcomes were generally consistent, but the correlation between gas pollutants exposure and risk for subtypes of preterm birth (ETB and VPTB) was stronger than that of the particulate matter.

Exposure to ambient air pollution was significantly associated with ETB and VPTB from the perspective of an entire pregnancy, and the correlation of gaseous pollutants was generally stronger than that of particulate matter. The pollutants with the most significant correlation of ETB and VPTB were SO_2_ and NO_2_, with HRs of 1.39 (95%CI: 1.29, 1.50) and 5.44 (95%CI: 4.75, 6.12), respectively. Particulate matters were significantly associated with ETB and VPTB as well, but their association was slightly lower than that of gaseous pollutants. In addition, PM_2.5_ and PM_10_ showed different relationships for different adverse pregnancy outcomes, and PM_2.5_ had a more significant correlation with ETB and VPTB than PM_10_ during the whole pregnancy.

As to the distribution of exposure windows, the critical trimester of various air pollutants for different adverse pregnancy outcomes were generally consistent. The critical exposure windows of PM_10_, PM_2.5_, and SO_2_ for ETB were found in the third trimester, with HRs of 1.06 (95%CI: 1.04, 1.09), 1.07 (95%CI: 1.04, 1.11), and 1.28 (95%CI: 1.20, 1.35), respectively. The exposure window of NO_2_ to ETB was also during middle and late pregnancy, but the HRs of NO_2_ in the second and third trimesters were close, reaching 1.10 (95%CI: 1.03, 6.17) and 1.09 (95%CI: 1.03, 1.15), respectively. Interestingly, the exposure windows for VPTB were in the first trimester. With each 10-μg/m^3^ increment in PM_10_, PM_2.5_, and SO_2_ exposure during the first trimester of gestation, the risk of VPTB increased by 1.14 (95%CI: 1.02, 1.26), 1.28 (95%CI: 1.04, 1.39), and 1.47 (95%CI: 1.15, 1.80), respectively. The correlation of exposure to NO_2_ was significant in the first and second trimesters, with the HRs 1.61 (95%CI: 1.29, 1.93) and 1.68 (95%CI: 1.38, 1.99), respectively.

**Figure 3:**
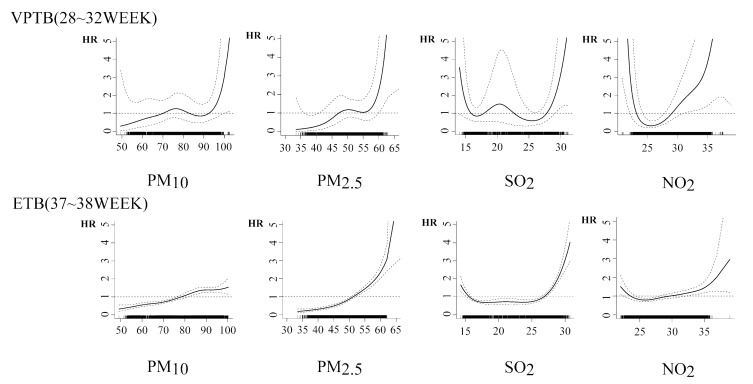
The concentration–response curves of different pollutants (µg/m^3^) during the whole pregnancy.

From the exposure–response curves (Figure 3), it was clearly observed that, with an increase of exposure concentration, the influence of gaseous pollutants and particulate matters on ETB and VPTB presented different trends. The curve of particulate matter showed a J-shape, which indicated that when the concentration of particulate matter reached a higher level, the increase would significantly increase the risk of VPTB and ETB. Conversely, when the concentration was at low levels, the increase in particulate concentration did not significantly affect the shortening of pregnancy. As an exception, the curve of PM_10_ and ETB generally maintained a simple gentle upward trend and did not reflect a typical J-shaped curve.

The curves of SO_2_ and NO_2_ indicated a U-shape, which was different from the particulate matters. When the concentrations were at low levels, the influence of gas pollutants on ETB and VPTB decreased with the increase of its concentration. However, at higher concentrations, the exposure concentrations of gaseous pollutants were positively correlated with the risk of ETB and VPTB. Meanwhile, the result of SO_2_ showed a slight fluctuation in the moderate level of the concentrations, while this phenomenon was not observed in the curve of NO_2_.

### 3.3. Stratified Analysis

An association between the ambient air pollution exposure and categorized preterm births stratified by maternal age, fetal gender, and maternal education is evaluated in Table 3. The maternal age subgroup was stratified by child-bearing age (<35 years old) and advanced maternal age (≥35 years old). The maternal education subgroup was stratified by low-level education (middle school or below) and high-level education (high school or above). The risk of exposure to all the four pollutants for ETB remained significant in all stratified groups. The HRs of ETB with a 10-μg/m^3^ increment in PM_10_, PM_2.5_, SO_2_, and NO_2_ were higher among women of advanced maternal age compared to those less than 35 years old and was mostly higher among those with low-level education than women with high-level education.

Different from the results of the stratified analysis on VPTB, women of child-bearing age and women who had a female fetus had higher risks of VPTB with increased exposure to ambient air pollution. NO_2_ still showed the strongest relevancy with VPTB, and HRs for a 10-μg/m^3^ increment in the pollutant concentration were 3.10 (95%CI: 2.13, 4.16), 4.33 (95%CI: 1.62, 7.04), and 3.73 (95%CI: 1.01, 6.45) for the subgroups of child-bearing age, female fetus, and high-level education, respectively. The correlation of some subgroups was not significant, which might be due to the limited sample sizes of VPTB.

## 4. Discussion

To our knowledge, this is the first study to investigate an association between ambient air pollution and preterm births in a small, medium-sized city of China—Shiyan. The findings of this study showed that maternal exposure to ambient air pollution is associated with an increased risk of shortened gestation in Shiyan. The influences of gaseous pollutants on ETB and VPTB were greater than that of particulate matters. The exposure to air pollution during the first trimester was more significantly associated with VPTB, while the exposure window of ETB was observed in the second half of pregnancy, especially in the third trimester. In addition, the exposure to ambient air pollution was associated to ETB in low-educated and elderly pregnancy women, but highly educated and children-bearing age pregnancy women were at high risk for VPTB.

Although previous studies have not reached a consensus about the key window period of ambient air pollution exposure for shortened gestation [14], there has been numerous emerging evidence suggesting that the ambient air pollution exposure in the second half of pregnancy exhibits a stronger connection with an increasing risk of adverse pregnancy outcomes. A systematic review and meta-analysis of 62 studies of air pollution and pregnancy outcomes found that the correlations of the third trimester exposures were generally the most precise and significant compared to the other stages of pregnancy, ranging from 4% per 1-ppm CO to 6% per 20-μg/m^3^ PM_10_ [20]. Another cohort study in Guangzhou showed that middle-to-late pregnancy was the most susceptible air pollutants exposure window for preterm births, which was consistent with the results of this study [21]. Some studies have even explored specifically the correlation of ambient air pollution exposure and adverse pregnancy outcomes only during the third trimester or a few weeks before delivery, which indicated a growing concern over the correlation of ambient air pollution exposure in the later stages of pregnancy [22]. The results of ETB in this study were consistent with the description of the relationship between air pollution exposure and PTB in previous studies. As a minor adverse pregnancy outcome relative to PTB advocated by ACOG [19], the relationship between ambient air pollution exposure and ETB should be generalized and echoed from previous conclusions on PTB. The adverse effects of ETB on newborns have been discussed in published studies [23], but the association between ambient pollutants exposure and ETB has still been rarely studied. The findings of this study complement the research for influencing the factors for ETB, and suggest that future studies in this field should pay more attention to no medically assisted deliveries at less than 39 weeks of gestation, which will improve the understanding of the association between ambient air pollution exposure and adverse pregnancy outcomes.

Compared with ETB, the risk of VPTB was more strongly associated with exposure to ambient air pollution during pregnancy. This study found that various air pollutant exposures during a whole pregnancy significantly increased the risk of VPTB, and the strongest correlation was observed in the first trimester. As an extreme subtype of preterm birth, VPTB has a significant correlation with the survival rate and long-term development of neonates [24]. A study in California reported that with a 10-μg/m^3^ increase in prenatal exposure to nitrogen oxides and PM_2.5_, the risk of VPTB increased by 227.62% and 240.77%, respectively [25]. Another research study conducted in Wuhan combined with the LUR model to assess the individual air pollutant exposure and, also, showed that an increase of particulate matter exposure concentration was significantly associated with an increased risk of VPTB [26], and the most significant correlation was obtained in the first trimester, as in this study. There are multiple biological and physiological mechanisms of how air pollutants may lead to deleterious health effects of the reproductive system during the first trimester. Early pregnancy is the key stage of embryo implantation and placenta formation, and many studies supported that adverse effects on the fetus during early pregnancy will seriously damage the health of the fetus and cause a series of pregnancy complications [27]. Inflammation and oxidative stress caused by maternal air pollutant exposure may result in aging of the placenta, which will increase the risk of extreme preterm births [5]. Moreover, PAHs (polycyclic aromatic hydrocarbons) carried by particulate matter can reach fetal organs through the placenta [28], causing fetal mtDNA damage or placental blood perfusion insufficiency in early pregnancy, ultimately leading to adverse pregnancy outcomes [6]. Therefore, combined with the evaluation of the influencing factors of VPTB in this study, a targeted improvement of the ambient air pollution exposure protection in early pregnancy may reduce the risk of the outcome of extreme preterm birth.

Besides the differences of the exposure windows, the correlations of different types of air pollutants also indicated a significant heterogeneity. Most current studies suggest that a high exposure concentration of particulate matters is significantly associated with adverse pregnancy outcomes, but the correlation of gas pollutants is controversial. Multiple studies have shown that SO_2_ exposure had the strongest correlation with shortening pregnancy [29], and the result of a series of studies in other countries reported that NO_2_ exposure increased the risk of preterm birth more significantly than particulate matters [30], which is consistent with our results. However, one study reported that the risk of preterm birth increased by 4.84% for each IQR increase in PM_2.5_ exposure, while the ER (Excess risks) of SO_2_ was 3.65% [31]. Another research study observed a significant association between NO_2_ exposure and preterm birth only at the end of pregnancy [22], and a natural experiment study even showed that PM_10_ and NO_2_ were both not significantly associated with preterm birth [32]. Previous studies have made reaching a consistent conclusion difficult on the correlation between the ambient air pollution and preterm birth, which are closely related to the increased exposure uncertainty caused by sparse spatiotemporal monitoring, a mismatch in the spatiotemporal scale/resolution, and the spatiotemporal misalignment of the environment and health dataset [33]. On the other hand, the population composition, climate characteristics, and pollutant sources of different areas also have a significant impact on the actual exposure at the individual level. As to this study, considering the main industries and ambient air pollution sources of the selected city, the study findings in Shiyan are reliable and in accordance with reality. According to a 2018 report by the municipal government, the auto industry is the most important industry in Shiyan, with a total output value of more than 21.62 billion dollars (140 billion CNY) and more than 200,000 employees. Meanwhile, according to the data from the Ministry of Ecology and Environment of the People’s Republic of China, gaseous pollutants (such as CO, NOx, and SO_2_) account for far more than particulate matters in the total vehicle emission pollution in China from 2010 to 2016 [34], which can help us further elucidate the differences in the correlations between various air pollutants and adverse reproductive health outcomes in Shiyan. In the case of outdoor air pollution exposure in Shiyan, the sources of ambient air pollution mainly come from the automobile manufacturing industry and vehicle emissions, meaning that gaseous pollutants occupy a large proportion in the composition of ambient air pollutants. In the case of personal exposure, the automobile industry in Shiyan is large in scale and requires many employees. For pregnant women who participate in the automobile manufacturing industry and need daily commuting, their exposure to SO_2_ and NO_2_ in the working environment will also exceed that of particulate matter. Thus, we observed a more significant association between gaseous pollutants and reproductive health in this study.

In the stratified analysis, we found that elderly pregnant women were at a high risk for ETB, which was consistent with the results of previous studies on adverse pregnancy outcomes in different maternal ages [35]. This result can be attributed to the aging of blood vessels in the reproductive system. The rate of placental vasoconstriction in older pregnant women is lower than younger pregnant women, while small particles of air pollutants can interfere with oxytocin secretion, further slowing the efficiency of placental vasoconstriction and leading to hypoxia in the fetus, thus increasing the risk of preterm birth [36]. Regarding the difference in ETB risk among the maternal education group, pregnant women with higher education levels were more likely to take the initiative to receive a health consultation and pregnancy care services, which was explained by previous studies [37]. We observed an opposite result from previous studies in the stratified analysis of VPTB, which may be due to the inconsistent proportions of each subtype of preterm birth in different study populations. In previous studies that analyzed the subtypes of preterm birth, the proportion of the VPTB population was about 1–3% [25,38], much higher than that in this study (0.48%). This difference indicates that the population composition of different regions will have a certain influence on the analysis of the relationship between the ambient air pollution and reproductive health, which probably provides a new research direction in this field.

This study has several strengths. Firstly, the pregnant women were selected as the research object from Shiyan Maternity and Child Health Hospital, which is the largest maternal and childcare hospital in Shiyan. More than 50 percent of Shiyan’s newborns are delivered here every year, suggesting that the correlation obtained from this study will be more representative. Secondly, the study population was recruited from a medium-sized city with a small floating population. As a result, most of the participants in the formal analysis were locals. The consistency of the object source ensures the homogeneity of the research objects and reduces the potential confusion by controlling the differences between individuals, thus increasing the statistical efficiency. Thirdly, previous studies in China were mainly focused on large cities and the provincial capital city, while this study further improved the description of the association between ambient air pollution exposure and the reproductive health of pregnant women in noncentral and medium-sized cities.

However, several limitations still exist in this study. Further caution is warranted in interpreting our findings, because our findings may be subject to bias, as we did not adjust for site-specific effects, nor did we account for the intracity variations in the air pollution exposure estimation, though there are variations in the air pollution exposure within our study area. We used the daily average of the air quality monitoring stations as the maternal daily air pollution exposure, but some studies have found that the correlation between air pollutant exposure and birth weight decreases with the increase of the distance interval used for the exposure estimation [39], which indicates that the uncertainty of exposure may be increased by aggregating the health data into a rough spatiotemporal range for an exposure analysis, taking the daily average data of fixed sites as the maternal daily exposure may lead to a personal level exposure misclassification [17]. Collecting the address information of all the participants, which is lacking in this study, dividing the study area into different exposure spaces according to the actual exposure concentration distribution for the hierarchical analysis may control the bias of an individual-level exposure assessment in ecological research. Meanwhile, because the hospital records for pregnant women do not contain information as to the health behaviors during pregnancy, such as smoking and alcohol consumption, we did not include these two important confounders in our model analysis. Although smoking and drinking are relatively rare among pregnant women in China [25], due to the influence of traditional Chinese culture and healthy pregnancy propaganda, the failure to control these two confounding factors may lead to a potential bias in this study. According to the relevant studies, because of the use of solid fuels [40] and more frequent cooking duties in the home [41], the indoor air pollution exposure of women in Chinese households may be different from the outdoor air pollution concentration, which may affect the interpretation of the relationship between the air pollutants and adverse pregnancy outcomes in this study.

## 5. Conclusions

In this study, the exposure to ambient air pollution during pregnancy is found to be significantly associated with the risk of ETB and VPTB in Shiyan, and the correlation of gaseous pollutants is stronger than particulate matter. The exposure to ambient air pollution during the first trimester is more significantly associated with VPTB, while the exposure window of ETB is observed in the second half of pregnancy. The risk may vary by maternal age and different maternal education levels. For local environmental administrative departments, our findings have important public health implications and provide an effective reference for the formulation of environmental protection policies.

## Figures and Tables

**Table 1:** The descriptive summary of the general characteristics of all the mothers.

Variables	TOTAL	ETB	PTB	VPTB
	(*n* = 13,111)	(*n* = 1246)	(*n* = 614)	(*n* = 64)
**Gestational age**	39.05 ± 1.48	37.47 ± 0.50	34.74 ± 1.99	30.23 ± 1.08
(mean ± SD, weeks)				
**Fetal gender**				
Male	6848(52.23%)	719(5.48%)	329(2.51%)	31(0.24%)
Female	6262(47.76%)	527(4.02%)	285(2.17%)	33(0.25%)
**Maternal age**				
Child-bearing age ^a^	11,768(89.76%)	1043(7.96%)	527(4.02%)	55(0.42%)
Advanced maternal age ^b^	1343(10.24%)	203(1.55%)	87(0.66%)	9(0.07%)
**Maternal education**				
Middle school or below	2085(15.90%)	222(1.70%)	123(0.94%)	8(0.06%)
High school or above	11,026(84.10%)	1024(7.81%)	491(3.74%)	56(0.42%)
**Gravidity**				
1	4390(33.48%)	323(2.46%)	171(1.30%)	18(0.14%)
2	3909(29.81%)	351(2.68%)	181(1.38%)	22(0.17%)
≥3	4812(36.70%)	572(4.36%)	262(2.00%)	24(0.18%)
**Parity**				
1	7241(55.23%)	553(4.22%)	292(2.23%)	30(0.23%)
2	5513(42.05%)	639(4.87%)	291(2.22%)	30(0.23%)
≥3	357(2.72%)	54(0.41%)	31(0.24%)	4(0.03%)

^a^ Pregnant women under the age of 35. ^b^ Pregnant women over 35 years old.

**Table 2:** HRs and 95% CIs of ETB, PTB, and VPTB for 10-µg/m^3^ increases of each pollutant in different trimesters. ^a^

Pollution	ETB		PTB		VPTB	
	HR (95%CI)	*p*	HR (95%CI)	*p*	HR (95%CI)	*p*
PM_10_						
Entire pregnancy	1.14(1.09,1.18)	<0.001	0.91(0.79,1.03)	0.123	1.43(1.21,1.65)	0.007
First trimester	1.01(0.98,1.04)	0.454	1.02(0.96,1.08)	0.556	1.14(1.02,1.26)	0.030
Second trimester	1.04(1.02,1.07)	0.002	0.99(0.94,1.05)	0.798	1.11(1.00,1.22)	0.381
Third trimester	1.06(1.04,1.09)	<0.001	0.90(0.84,0.95)	<0.001	1.01(0.90,1.12)	0.192
PM_2.5_						
Entire pregnancy	1.26(1.17,1.35)	<0.001	0.98(0.92,1.04)	0.516	1.78(1.36,2.20)	0.002
First trimester	1.01(0.97,1.05)	0.770	1.01(0.97,1.05)	0.616	1.22(1.04,1.39)	0.037
Second trimester	1.05(1.01,1.09)	0.011	1.00(0.96,1.04)	0.943	1.08(0.91,1.24)	0.072
Third trimester	1.07(1.04,1.11)	<0.001	0.94(0.91,0.98)	0.002	0.89(0.72,1.07)	0.911
SO_2_						
Entire pregnancy	1.39(1.29,1.50)	<0.001	0.89(0.74,1.04)	0.134	2.07(1.59,2.55)	0.003
First trimester	1.18(1.11,1.26)	<0.001	0.94(0.83,1.05)	0.291	1.47(1.15,1.80)	0.020
Second trimester	1.13(1.05,1.21)	0.004	0.93(0.80,1.05)	0.233	1.32(0.98,1.66)	0.114
Third trimester	1.28(1.20,1.35)	<0.001	0.82(0.69,0.94)	0.001	1.30(0.98,1.63)	0.107
NO_2_						
Entire pregnancy	1.37(1.23,1.51)	<0.001	1.03(0.82,1.23)	0.812	5.44(4.75,6.12)	<0.001
First trimester	1.04(0.97,1.12)	0.250	1.02(0.91,1.12)	0.762	1.61(1.29,1.93)	0.003
Second trimester	1.10(1.03,1.17)	0.009	1.02(0.92,1.12)	0.685	1.68(1.38,1.99)	0.001
Third trimester	1.09(1.03,1.15)	0.009	0.93(0.83,1.03)	0.172	0.86(0.54,1.17)	0.337

^a^ HR adjusted for maternal age, maternal education, gravidity, parity, and fetal gender. Abbreviation: CI, confidence interval; ETB, ETB (37 to 38 weeks); PTB, preterm birth (less than 37 weeks); and VPTB, very preterm birth (28–32 weeks).

**Table 3:** HR of ETB and VPTB for a 10-µg/m^3^ increase of each pollution over an entire pregnancy stratified by the maternal age, fetal gender, and maternal education.

Subgroup	PM_10_		PM_2.5_		SO_2_		NO_2_	
	HR (95%CI) ^a^	*p*	HR (95%CI)	*p*	HR (95%CI)	*p*	HR (95%CI)	*p*
**ETB**								
Maternal age								
Child-bearing age^b^	1.12(1.06,1.18)	<0.001	1.43(1.27,1.59)	<0.001	1.33(1.20,1.45)	<0.001	1.44(1.18,1.71)	<0.001
Advanced maternal age^c^	1.23(1.07,1.39)	0.001	1.53(1.14,1.93)	0.003	1.39(1.10,1.70)	0.001	1.70(1.01,2.44)	0.014
Fetal gender								
Male	1.13(1.06,1.20)	<0.001	1.25(1.11,1.40)	<0.001	1.36(1.18,1.56)	<0.001	1.31(1.09,1.58)	0.005
Female	1.14(1.06,1.22)	<0.001	1.26(1.10,1.45)	0.001	1.41(1.20,1.66)	<0.001	1.43(1.14,1.78)	0.002
Maternal education								
Middle school or below	1.23(1.09,1.38)	0.001	1.51(1.21,1.88)	0.001	1.63(1.26,2.10)	<0.001	1.82(1.29.2.55)	0.001
High school or above	1.11(1.06,1.17)	<0.001	1.21(1.09,1.33)	<0.001	1.33(1.18,1.49)	<0.001	1.27(1.08,1.49)	0.003
**VPTB**								
Maternal age								
Child-bearing age	1.39(1.11,1.68)	<0.001	1.69(1.02,2.40)	<0.001	2.08(1.45,2.75)	<0.001	3.10(2.13,4.16)	<0.001
Advanced maternal age	0.96(0.89,1.47)	0.039	0.91(0.76,2.00)	0.04	0.87(0.71,1.94)	0.088	0.91(0.71,2.71)	0.522
Fetal gender								
Male	1.18(0.88,1.59)	0.262	1.15(0.67,2.00)	0.612	1.69(0.86,3.31)	0.131	3.14(1.25,7.88)	0.015
Female	1.79(1.26,2.53)	0.001	3.03(1.54,5.94)	0.001	2.58(1.28,5.19)	0.008	4.33(1.62,7.04)	<0.001
Maternal education								
Middle school or below	0.70(0.40,1.22)	0.203	0.38(0.14,1.05)	0.062	0.39(0.09,1.75)	0.219	0.41(0.06,2.81)	0.363
High school or above	1.66(1.29,2.14)	<0.001	2.51(1.54,4.10)	<0.001	2.79(1.63,4.79)	<0.001	3.73(1.01,6.45)	<0.001

^a^ Cox proportional hazards regression model, adjusted for maternal age, maternal education, gravidity, parity, and the gender of the baby. ^b^ Pregnant women under the age of 35. ^c^ Pregnant women over 35 years old.

## Appendix A

**Table A1:** Descriptive statistics for the mean and standard deviation of air pollution concentrations (μg/m^3^).

Air Pollutants	Total	Reference *	ETB	PTB	VPTB
	*n* = 13,111	*n* = 11,251	*n* = 1246	*n* = 614	*n* = 64
**Entire pregnancy**					
PM_10_	80.62 ± 12.49	80.49 ± 12.36	82.1 ± 12.57	80.15 ± 14.42	85.43 ± 16.31
PM_2.5_	51.57 ± 6.56	51.51 ± 6.45	52.34 ± 6.78	51.12 ± 7.94	53.73 ± 8.87
SO_2_	21.93 ± 5.36	21.87 ± 5.32	22.66 ± 5.54	21.54 ± 5.53	23.84 ± 6.66
NO_2_	28.72 ± 3.90	28.66 ± 3.83	29.21 ± 4.18	28.78 ± 4.50	31.24 ± 5.90
**First trimester**					
PM_10_	84.87 ± 19.70	84.85 ± 19.60	85.00 ± 20.31	85.04 ± 20.31	89.58 ± 21.18
PM_2.5_	53.90 ± 13.61	53.88 ± 13.57	53.93 ± 13.75	54.11 ± 13.88	57.35 ± 14.41
SO_2_	23.32 ± 7.17	23.27 ± 7.14	23.97 ± 7.51	22.91 ± 7.04	25.19 ± 8.12
NO_2_	29.42 ± 7.77	29.39 ± 7.75	29.68 ± 7.97	29.48 ± 7.91	32.23 ± 8.93
**Second trimester**					
PM_10_	79.79 ± 21.30	79.64 ± 21.30	81.25 ± 20.45	79.58 ± 23.00	84.4 ± 21.15
PM_2.5_	50.99 ± 14.31	50.91 ± 14.29	51.86 ± 14.00	50.81 ± 15.21	52.57 ± 12.59
SO_2_	21.59 ± 6.57	21.57 ± 6.55	21.98 ± 6.76	21.23 ± 6.56	22.90 ± 6.83
NO_2_	28.13 ± 7.74	28.05 ± 7.72	28.74 ± 7.77	28.29 ± 8.08	31.46 ± 8.34
**Third trimester**					
PM_10_	77.16 ± 22.46	77.01 ± 22.35	79.88 ± 23.19	74.32 ± 22.37	77.52 ± 21.90
PM_2.5_	49.74 ± 14.90	49.71 ± 14.82	51.18 ± 15.41	47.51 ± 14.87	47.39 ± 15.75
SO_2_	20.99 ± 7.05	20.92 ± 6.94	22.08 ± 7.79	20.03 ± 7.18	22.35 ± 8.71
NO_2_	28.59 ± 7.99	28.54 ± 7.90	29.23 ± 8.68	28.23 ± 8.05	27.76 ± 9.00

* Gestation age more than 38 weeks.
